# Biogeochemical Changes During Bio-cementation Mediated by Stimulated and Augmented Ureolytic Microorganisms

**DOI:** 10.1038/s41598-019-47973-0

**Published:** 2019-08-08

**Authors:** Michael G. Gomez, Charles M. R. Graddy, Jason T. DeJong, Douglas C. Nelson

**Affiliations:** 1 Department of Civil and Environmental Engineering, University of Washington, Seattle, WA 98195 USA; 2 Department of Microbiology and Molecular Genetics, University of California, Davis, CA 95616 USA; 3 Department of Civil and Environmental Engineering, University of California, Davis, CA 95616 USA

**Keywords:** Biogeochemistry, Applied microbiology, Civil engineering

## Abstract

Microbially Induced Calcite Precipitation (MICP) is a bio-mediated cementation process that can improve the engineering properties of granular soils through the precipitation of calcite. The process is made possible by soil microorganisms containing urease enzymes, which hydrolyze urea and enable carbonate ions to become available for precipitation. While most researchers have injected non-native ureolytic bacteria to complete bio-cementation, enrichment of native ureolytic microorganisms may enable reductions in process treatment costs and environmental impacts. In this study, a large-scale bio-cementation experiment involving two 1.7-meter diameter tanks and a complementary soil column experiment were performed to investigate biogeochemical differences between bio-cementation mediated by either native or augmented (*Sporosarcina pasteurii)* ureolytic microorganisms. Although post-treatment distributions of calcite and engineering properties were similar between approaches, the results of this study suggest that significant differences in ureolysis rates and related precipitation rates between native and augmented microbial communities may influence the temporal progression and spatial distribution of bio-cementation, solution biogeochemical changes, and precipitate microstructure. The role of urea hydrolysis in enabling calcite precipitation through sustained super-saturation following treatment injections is explored.

## Introduction

Microbially induced calcite precipitation (MICP) is a bio-mediated cementation process that can bind soil particles at contact locations, coat soil particle surfaces, and reduce void space in porous media^1,2^. Most commonly this process is accomplished through the use of bacteria containing urease enzymes, which catalyze a hydrolysis reaction that degrades urea and generates ammonia and carbonic acid (Eq. (1))^3^. When surrounding solution pH is not highly basic, a fraction of produced ammonia ions will undergo an equilibrium reaction with water resulting in the production of ammonium and hydroxide ions (Eq. (2)). This hydroxide production drives the de-protonation of produced carbonic acid to form increased concentrations of bicarbonate and carbonate ions (Eq. (3)). When soluble calcium ions are provided from treatment injections or groundwater, soil solutions may become super-saturated and calcite precipitation can occur (Eq. (4)).

1 $${(N{H}_{2})}_{2}CO+\,2{H}_{2}O\to 2N{H}_{3}+{H}_{2}C{O}_{3}$$

2 $$N{H}_{3}+{H}_{2}O\leftrightarrow N{H}_{4}^{+}+O{H}^{-}$$

3 $${H}_{2}C{O}_{3}\leftrightarrow HC{O}_{3}^{-}+{H}^{+}\leftrightarrow C{O}_{3}^{-2}+2{H}^{+}$$

4 $$C{a}^{+2}+C{O}_{3}^{-2}\leftrightarrow CaC{O}_{3(solid)}$$

Potential applications of MICP include geotechnical soil improvement for mitigation of earthquake-induced soil liquefaction^4,5^, immobilization of groundwater contaminants^6^, sealing of rock fractures for waste storage and carbon sequestration^7^, modification of flow in porous media for contaminant transport and petroleum recovery^8,9^, and healing of cracked concrete materials^10^.

Researchers have primarily relied on the injection of specialized laboratory-cultured microorganisms, such as *Sporosarcina pasteurii*, to complete bio-cementation through an augmentation approach^2,11–13^. Although effective, researchers have recently demonstrated that native soil microorganisms can be enriched in natural soil materials to complete urea hydrolysis through a stimulation approach^14–24^. The use of native ureolytic microorganisms may reduce treatment costs and environmental impacts by eliminating the materials and energy associated with bacterial cultivation and transportation and potential ecological impacts related to the introduction of high densities of non-indigenous bacterial species into soil ecosystems^22^. Furthermore, the stimulation process may improve spatial uniformity of ureolytic microorganisms, and therefore enhance bio-cementation uniformity, by eliminating colloidal filtration of bacterial cells that can occur during high cell density augmentation injections and result in log-linear decreases in bacterial cells with distance from the injection location^25^.

In this study, a large-scale tank experiment was completed to investigate biogeochemical differences between bio-cementation mediated by native ureolytic microorganisms and augmented *S*. *pasteurii*. Although the final engineered improvement was similar between approaches^22^, spatial and temporal differences in biogeochemical reaction rates were observed, which may have important implications for field-scale applications wherein complexities related to subsurface hydraulic gradients, soil heterogeneity, and differences in injection rates may occur. Two 1.7 meter diameter tanks containing a poorly-graded sand were treated using three wells in a triangular pattern and treatments were applied in three phases over 14 days^22^. Initial hydraulic properties in tanks were evaluated using passive tracer testing prior to treatment injections. In both tanks, biological treatments were first completed to establish either native or *S*. *pasteurii* microorganisms and eight cementation injections were subsequently performed. Following injections, temporal and spatial changes in aqueous calcium, bicarbonate, carbonate, ammonia, ammonium, and urea concentrations as well as ionic strength, solution pH, total alkalinity, and saturation ratio values were examined. Changes in precipitation spatial distributions and the influence of reactions during injections were evaluated using direct calcite, aqueous chemical, and non-destructive shear wave velocity (V_s_) measurements. To further characterize biological and chemical differences between approaches, a complementary soil column experiment was performed under conditions similar to the tank experiments and ureolysis rates and cell densities were evaluated in time. Following all treatments, precipitate microstructures were examined using Scanning Electron Microscope (SEM) imaging. Differences in ureolysis and precipitation kinetics, aqueous chemical changes, and precipitate spatial distributions and microstructures between tank experiments were specifically investigated to improve our understanding of the implications of using native ureolytic microorganisms for the bio-cementation process as an alternative to a more traditional augmentation approach.

## Methods

A summary of relevant materials and methods are provided here, with additional details related to the large-scale tank experiment presented in a separate manuscript^22^.

### Tank specimens

**Figure 1:**
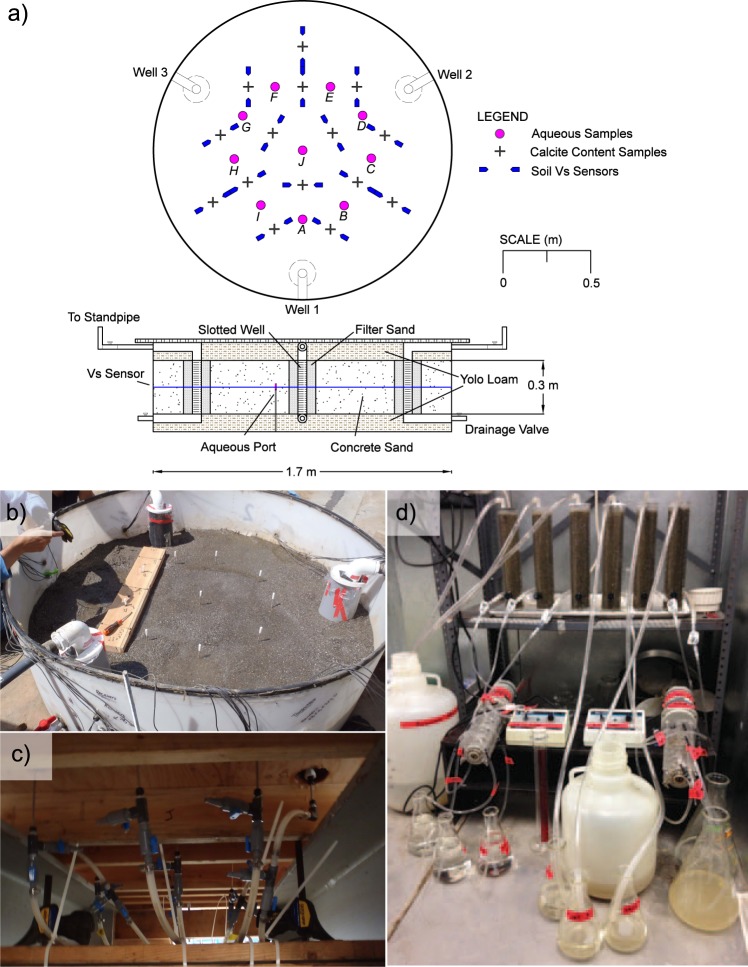
(**a**) Plan and cross-section views of tank specimens including soil layers and measurement locations, (**b**) aqueous sampling ports during soil placement, (**c**) sample collection valves, and (**d**) soil columns and treatment application system.

Two 1.7 m diameter, 0.5 m high cylindrical tanks were prepared with a single 0.3 m thick layer of dry-pluviated poorly-graded sand (D_10_ = 0.18 mm, D_50_ = 1.07 mm, fines content = 3%, relative density ≈ 43 to 51%) at mid-height and two 0.1 m thick layers of low-plasticity clay on top and bottom. Three treatment wells were positioned in a triangular pattern at well-to-well spacings of 1.2 meters and were slotted only within the sand layer targeted for bio-cementation. Coarser sand (D_10_ = 0.95 mm, D_50_ = 1.34 mm, fines content < 1%, relative density ≈ 60%) was placed in 5.1 cm annuli around slotted well sections to inhibit potential erosion of finer sand into wells. The total pore volume (PV) of the sand treatment layer was 214 L. Impermeable plastic liners separated soil layers to mitigate interactions between solutions and clay minerals. Following soil placement, a top cap was applied to seal tanks and prevent hydraulic piping between wells. Figure 1a presents cross-section and plan-view schematics of tank specimens including well locations, layer thicknesses, and measurement locations.

### Passive tracer testing

Prior to all treatments, tanks were saturated with deionized water and passive tracer testing was completed to verify that solution transport was similar between tanks. During tracer testing, 2.25 PV of 15 mM NaBr solution was injected, followed by a 2.25 PV injection of deionized water along well-to-well alignments while measuring solution conductivity at effluent wells. Tracer testing was completed from Well 1 to Well 2 (W1 to W2) and from Well 2 to Well 3 (W2 to W3) in both tanks to evaluate transport along alignments that would receive cementation injections. Conductivity measurements were correlated to Br^−^ concentrations using calibration relationships.

### Treatment solution injections

Following passive tracer testing, treatments were completed in three phases over a total of 14 days to: (1) establish microorganisms capable of ureolysis, (2) initiate calcite precipitation, and (3) remove non-calcite process by-products. For Day 1 to 4, treatments were completed to either establish native (stimulation) or *S*. *pasteurii* (augmentation) ureolytic soil microorganisms uniformly throughout respective tanks. In the stimulation tank, stimulation solution treatments were completed once daily and involved a series of three 0.5 PV stop-flow injections performed in a cyclic, uniform manner. The stimulation solution contained 350 mM urea, 12.5 mM NH_4_Cl, 42.5 mM C_2_H_3_NaO_2_, and 0.1 g/L yeast extract (pH ≈ 7.3). During injections, one well served as an injection well, a second well served as a production well, and a third well remained passive. A consistent injection order from W1 to W2, W2 to W3, and W3 to W1 was used with the starting alignment alternated daily to mitigate potential bias. Injections were completed at an average flow rate of 0.85 PV/hr using a constant head difference of 0.6 m selected to transport reactive constituents at low Damköhler and high Péclet number conditions immediately between well pairs.

The augmentation tank received no treatment injections from Day 1 to 3, however, on Day 4 an augmentation solution identical to the stimulation solution but with *S*. *pasteurii* cells at 3.5 × 10^7^ cells/mL was applied in three 0.5 PV injections. An additional augmentation solution volume of 208 L was re-circulated for six additional 0.5 PV injections and was retained for 26.5 hours. On Day 5, immediately prior to the initiation of cementation injections, a pre-cementation flush treatment involving three 0.5 PV stimulation solution injections was performed in both tanks. Following this flush, daily non-uniform cementation solution injections were applied to both tanks from Day 5 to 12. Cementation solutions were identical to the stimulation solution but included 250 mM CaCl_2_. For Day 5 to 9, both tanks received a single 0.75 PV cementation solution injection once daily from W1 to W2. For Day 10 to 12, the injection direction was changed and identical cementation injections were completed from W2 to W3. Following cementation injections, an artificial groundwater (AGW) solution^26^ was applied in three 2.25 PV injections to remove remaining biomass, ammonium, and soluble non-calcite salts. The AGW solution contained 40 μM KNO_3_, 450 μM MgSO_4_, 1.75 mM CaCl_2_, 40 μM NaNO_3_, 1.1 mM NaHCO_3_, and 60 μM KHCO_3_.

### Aqueous sampling

Aqueous sampling ports were used to obtain solution samples at various times during treatment at ten locations within each tank (Fig. 1a). Ports constructed with stainless-steel tubes with 130 μm filter tips open at mid-depth were pre-installed within the sand layer. Figure 1b presents an image of sampling ports within a tank specimen during soil placement and Fig. 1c presents an image of port valves used to obtain samples. Aqueous samples of approximately 40 mL were collected from port locations on all treatment days before and immediately after injections. On alternating treatment days, samples were also collected two and four hours after injections. Prior to sample collection, fluid volumes of ≈20 mL were drained from ports and discarded. All samples were frozen immediately and stored at −20 °C.

### Aqueous measurements

Solution pH measurements were completed during sample collection and immediately prior to total alkalinity, ammonium, and calcium measurements (after freeze-thaw) using a pH electrode system. pH electrodes were calibrated using a three-point buffer system (4.01, 7.00, 10.00) and measurements had ± 0.01 pH unit accuracy. pH measurements obtained before and after freeze-thaw differed by no more than 0.05 pH units. Urea measurements were completed using a modified colorimetric urea assay^27^. In the assay, a colorimetric reagent consisting of 3.2% (w/v) p-Dimethylaminobenzadehyde, and 24% (v/v) HCl in 99.8% ethanol was added to dilute samples and absorbance was measured at 422 nm using a spectrophotometer. The method detection limit (MDL) for the assay was 5 mM urea. Total ammonium (Total-NH_4_^+^) measurements were completed using a flow injection analyzer system using a modified method^28^. In the method, dilute samples were mixed with 936 mM sodium salicylate, 3.8 mM sodium nitroprusside, 45.1 mM sodium hypochlorite, 130 mM sodium phosphate dibasic heptahydrate, 70 mM EDTA, 1.25 M NaOH, and samples were heated to induce a colorimetric reaction. Absorbance was measured at 660 nm using a spectrophotometer and the MDL for the assay was 3 μM total-NH_4_^+^. NH_4_^+^ and NH_3_ concentrations were determined from solution pH, total-NH_4_^+^ measurements, and the pKa for NH_4_^+^ of 9.24^29^. Calcium (Ca^+2^) measurements were completed using inductively coupled plasma atomic emission spectrometry with a ICP-OES spectrometer following Method 200.7 from the U.S. EPA^30^. The MDL for this measurement was 1.25 μM Ca^+2^. Total alkalinity (A_T_) measurements were completed using an acid-titration procedure similar to ASTM D1067-16^31^ wherein additions of 12.5 mM H_2_SO_4_ were performed in the presence of a bromocresol green indicator. Proton acceptors were considered to be bases formed from weak acids with dissociation constants less than 10^−4.5^ at 25 °C and zero ionic strength^32^. Solution A_T_ in collected samples were attributed to HCO_3_^−^, CO_3_^−2^, OH^−^, and NH_3_ concentrations while neglecting other trace proton acceptors from soil solution (Eq. (5)). The MDL for this measurement was 0.04 meq/L. NH_3_, A_T_, and solution pH determined at the time of chemical analyses were used to determine CO_3_^−2^ and HCO_3_^−^ concentrations from the pKa_2_ for HCO_3_^−^/CO_3_^−2^ of 10.3^29^ (Eqs (6) and (7)).

5 $${A}_{T}=[HC{O}_{3}^{-}]+\,2[C{O}_{3}^{-2}]+[O{H}^{-}]+[N{H}_{3}]$$

6 $$[{{\rm{HCO}}}_{3}^{-}]=\frac{[{{\rm{H}}}^{+}]({{\rm{A}}}_{{\rm{T}}}-[{{\rm{OH}}}^{-}]+[{{\rm{NH}}}_{3}])}{[{{\rm{H}}}^{+}]+2[{10}^{-pK{a}_{2}}]}$$

7 $$[C{O}_{3}^{+2}]=\frac{[{10}^{-pK{a}_{2}}][HC{O}_{3}^{-}]}{[{H}^{+}]}$$

### Saturation ratio determination

Solution ionic strength was calculated using Ca^+2^, NH_4_^+^, HCO_3_^−^, and CO_3_^−2^ measurements and other known concentrations from solution additions. Activity coefficients for calcium (γ_Ca+2_) and carbonate (γ_CO3-2_) species were determined using the Extended Debye-Hückel equation for all samples with I of less than 0.1 M. The ion size parameter *a* was assumed to be 6 Å for calcium and 5 Å for carbonate^33^, and the temperature and solution constants *A* and *B* were assumed to be 0.5 and 0.33 × 10^8^, respectively, for water at 25 °C^34^. For samples with I values between 0.1 and 0.7 M, the Davies activity coefficient equation^35^ was used. Solution saturation ratios (Ω) with respect to calcite were determined following (Eq. (8)) by dividing the ion activity product (IAP), or product of Ca^+2^ and CO_3_^−2^ activities, by the solubility product (K_sp_) for calcite mineral, which was assumed to be 10^−8.48^ at 1 atm and 25 °C^29^.

8 $${\rm{\Omega }}=\frac{IAP}{{K}_{sp}}=\frac{{\gamma }_{C{a}^{+2}}[C{a}^{+2}]{\gamma }_{C{O}_{3}^{-2}}[C{O}_{3}^{-2}]}{{K}_{sp}}$$

### Shear wave velocity measurements

Shear wave velocity (V_s_) measurements were completed using bender element sensors to monitor changes in soil small-strain shear stiffness resulting from calcite precipitation. Measurements were completed at 15 locations at mid-depth within each tank (Fig. 1a). Transmitting sensors were excited using a 24 V 100 Hz square wave and were measured at a sampling frequency of 51.2 kHz. Measurements were obtained at all locations immediately prior to daily treatment injections.

### Direct calcite content measurements

Following the treatment program, soil samples were obtained between bender element sensors using sampling tubes. Samples from mid-depth were dried and mixed to obtain representative samples and calcite contents were quantified in accordance with ASTM D4373^36^ using HCl dissolution and a pressure chamber. Relationships between chamber pressures and reagent-grade CaCO_3_ masses were used to determine soil calcite contents.

### Scanning electron microscope images

Scanning electron microscope images were completed with a Hitachi S-4100 field emission SEM at an acceleration voltage of 2 kV and magnification of 100x. Prior to imaging, cemented samples were oven dried for at least 5 days. Calcite crystal sizes were estimated using ImageJ software^37^.

### Soil column experiment

A complementary soil column experiment was completed to further characterize temporal changes in microbial urea hydrolysis and aqueous cell densities during treatment. Six soil columns (30.5 cm long, 5 cm inside diameter) were prepared using the same sand and were separated into two sets of three columns, which received treatments identical to either the stimulation or augmentation tank. Similar to the tank experiment, augmentation columns received an augmentation solution that was identical to the stimulation solution but contained *S*. *pasteurii* cells at 4.3 × 10^7^ cells/mL and was recirculated. All treatment injections were applied to match the average hydrodynamic transport rates, pore water velocities, and residence times between treatments in the tank experiments as closely as possible. Daily injections of 2 PV were completed at a flow rate of 1.8 L/hr, consistent with average pore-water velocities at locations directly along well-to-well alignments. One soil column from each set was destructively sampled after 1, 4, and 8 cementation injections to examine changes in precipitate microstructure in time. Soil columns and the solution application system are shown in Fig. 1d.

### Direct cell count measurements

Solution volumes of 0.5 to 1.0 mL were collected from columns before daily injections to monitor changes in aqueous cell densities. Total direct cell counts were completed by the acridine orange staining epifluorescence method^38^ and were corrected for background using sterile saline.

## Results and Discussion

### Pre-treatment conditions

**Figure 2:**
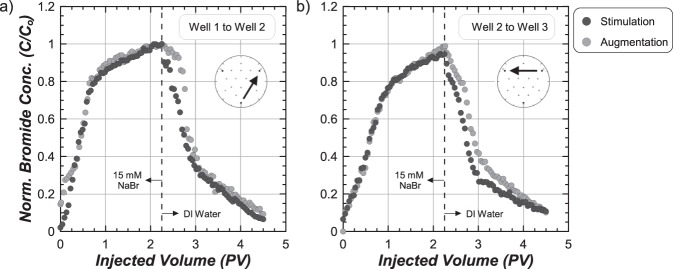
Normalized bromide concentrations (C/Co) versus injected pore volumes (PV) from passive tracer testing completed from (**a**) Well 1 to Well 2 and (**b**) Well 2 to Well 3.

Figure 2a,b present normalized bromide concentration (C/C_o_) measurements versus injected volume from passive tracer tests completed from W1 to W2 and W2 to W3 in both tanks. W1 to W2 breakthrough curves were nearly identical between tanks with C/C_o_ of 0.8 obtained after ≈0.75 PV and 1.0 obtained after ≈2.25 PV. Following the subsequent deionized water injection, similar reductions in C/C_o_ values were observed with C/C_o_ values near 0.4 after an additional 0.75 PV. W2 to W3 curves were also similar between tanks. Again, C/C_o_ values near 1.0 were obtained after ≈2.25 PV and reductions in C/C_o_ values near 0.35 were obtained in both tanks following an additional 0.75 PV of deionized water. Although only small differences were observed between tanks for the same alignment, significant differences were observed between W1 to W2 and W2 to W3. In both specimens, C/C_o_ values consistently increased more rapidly with injected volume along W1 to W2. Breakthrough curves from W2 to W3 were consistent with measured soil porosities and expected trends from numerical models^39^, suggesting that the W1 to W2 alignment may have been initially partially-saturated. Despite these differences, similar breakthrough curves for each well alignment suggested that advective-dispersive solution transport between tanks was comparable prior to treatments.

### Urea degradation

**Figure 3:**
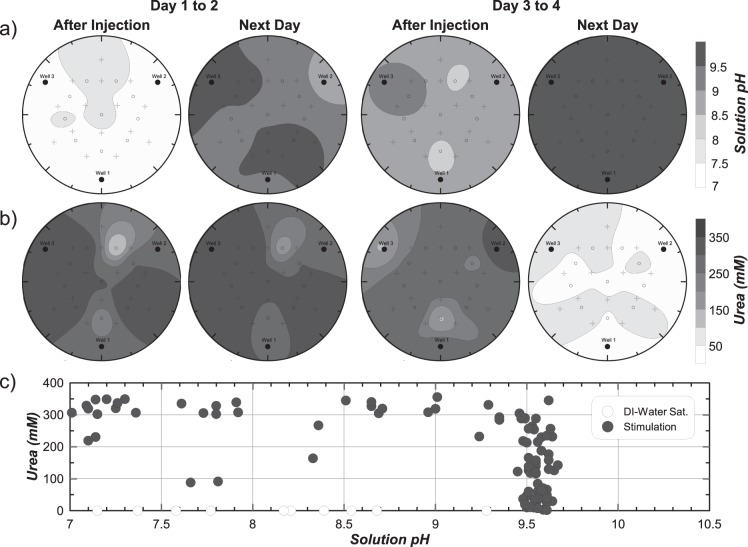
Spatial contours of (**a**) solution pH and (**b**) aqueous urea concentrations within the stimulation tank from Day 1 to Day 2 and Day 3 to Day 4, and (**c**) the relationship between solution pH and urea concentration for all measurements from the stimulation tank during stimulation.

Figure 3a,b present inverse-distance interpolated spatial contours of solution pH and aqueous urea concentrations, respectively, within the stimulation tank immediately after injection and following a ≈22-hour retention period for Day 1 to 2 and Day 3 to 4. Following the first stimulation solution injection on Day 1, pH values were between 7.0 and 7.5 and urea concentrations were near 300 mM at most locations. After a 22-hour residence period, solution pH values increased to above 8.5 at most locations with several locations reaching pH values near 9.5. Despite this significant pH rise, urea degradation was not detected. From Day 3 to 4, significant increases in solution pH from values near 8.5 after injection to near 9.5 after 22 hours were again observed. This time, however, urea concentrations near 300 mM after injection were degraded to near and below 50 mM. This significant urea degradation suggested that native ureolytic microorganisms were completing urea hydrolysis in a spatially uniform manner in the stimulation tank by Day 4.

Although large increases in solution pH were observed following both injections, urea degradation between days differed significantly. Corresponding urea concentration and solution pH measurements obtained from the stimulation tank from Day 1 to 5 were plotted in Fig. 3c to evaluate the relationship between urea degradation and pH rise. Following saturation with deionized water, urea concentrations were not detectable, and pH varied between 7.1 and 9.3. Following stimulation injections, many locations had urea concentrations near 300 mM and pH values that increased from near 7.0 to 9.5 without large changes in urea concentrations. Large changes in solution pH without significant urea degradation has been observed in other stimulation experiments^23^ and results from limited initial pH buffering in stimulation solutions with subsequent buffering by NH_3_/NH_4_^+^ after sufficient urea hydrolysis. At a near-constant pH of 9.5, urea concentrations decreased from near 300 mM to 0 mM, suggesting that monitoring of solution pH changes during stimulation may not provide an effective method for assessing ureolysis.

**Figure 4:**
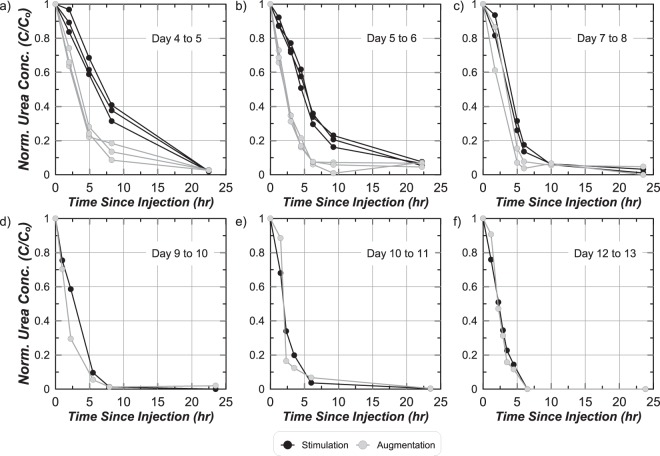
Normalized aqueous urea (C/C_o_) concentrations with time since injection for stimulation and augmentation soil columns for (**a**) Day 4 to 5, (**b**) Day 5 to 6, (**c**) Day 7 to 8, (**d**) Day 9 to 10, (**e**) Day 10 to 11, and (**f**) Day 12 to 13.

Figure 4a,f present normalized urea concentration (C/C_o_) measurements versus time after treatment injections for all soil columns for select treatment days during stimulation/augmentation and cementation phases. Measurements were normalized by urea concentrations measured immediately after injections (C_o_). On Day 4 to 5, stimulation columns received the last stimulation treatment and augmentation columns were augmented. Urea degradation was significantly faster in the augmentation columns with ≈75% urea degraded after 5 hours and full urea degradation after 22 hours (Fig. 4a). In the stimulation columns ≈35% was degraded over 5 hours, yet full degradation was still achieved after 22 hours. Ureolysis rates increased in both column sets following the first cementation injection on Day 5 to 6, but remained faster in the augmentation columns with ≈82% degraded within 5 hours and full degradation after ≈10 hours (Fig. 4b). From Day 7 to 8, ureolysis rates further increased with ≈90% and ≈70% urea degradation occurring within 5 hours in augmentation and stimulation columns, respectively (Fig. 4c). For all cementation treatments after Day 9 (Fig. 4d–f), ureolysis rates were almost identical between augmentation and stimulation columns and continued to increase in time. From Day 12 to 13, the highest ureolysis rates were observed with full urea degradation occurring within ≈6 hours. Increases in ureolysis rates during cementation may have resulted from further enrichment of native ureolytic microorganisms in both column sets^24^, increases in attached microbial populations, or potential lysis of cells and release of free urease.

### Aqueous cell growth

Aqueous total cell densities were measured in stimulation and augmentation columns using samples obtained before daily injections. During the first 3 days, no injections were completed in the augmentation columns and native cell densities remained near 5 × 10^6^ cells/mL. Following augmentation on Day 4, however, cell densities in the augmentation columns increased by nearly an order of magnitude to 3 × 10^7^ cells/mL. At this same time in stimulation columns, cell densities gradually increased and a comparable cell density near 4.5 × 10^7^ cells/mL was obtained on Day 4. During cementation, higher cell densities were observed in stimulation columns from Day 5 to 8 despite having lower ureolysis rates (Fig. 4). Higher cell densities that did not coincide with higher ureolysis rates in the stimulation columns may be attributed to the presence of other non-ureolytic cells, differences in ureolysis rates between strains, and changes in cell densities attached to soil particles (not measured). From Day 8 to 13, similar cell densities were measured in both augmentation and stimulation columns near 6 × 10^6^ cells/mL.

### Aqueous chemical changes

**Figure 5:**
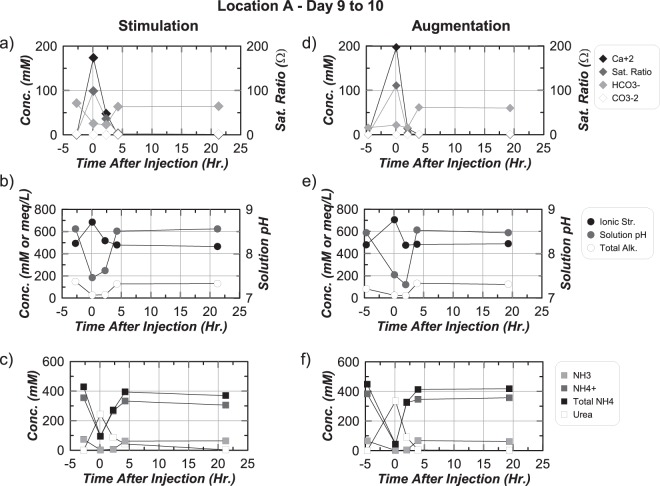
Measurements of (**a**,**d**) Ca^+2^, Ω, HCO_3_^−^, CO_3_^−2^, (**b**,**e**) ionic strength, solution pH, total alkalinity, (**c**,**f**) NH_3_, NH_4_^+^, total-NH_4_^+^, and urea in time at location A in both tanks from Day 9 to Day 10.

Figure 5a through 5f present aqueous chemical measurements in time obtained from Location A (Fig. 1) during the Day 9 to 10 cementation injection in stimulation and augmentation tanks. This cementation injection was the last of five completed from W1 to W2. Location A is presented due to its proximity to injection W1 and location within the main well-to-well alignment wherein other complicating factors related to transport were minimized. In the stimulation tank immediately following injection, Ca^+2^ concentrations increased from near 0 to 175 mM, saturation ratios (Ω) increased from near 1 to 100, HCO_3_^−^ concentrations reduced from 75 to 25 mM, and CO_3_^−2^ concentrations reduced from 1.3 to 0.03 mM (Fig. 5a). After 2 hours, Ca^+2^ concentrations were reduced by ≈125 mM, Ω decreased to near 40, and HCO_3_^−^ (23 mM) and CO_3_^−2^ (0.05 mM) concentrations remained nearly constant. Limited changes in CO_3_^−2^ concentrations likely resulted from the simultaneous production and consumption of CO_3_^−2^ from ureolysis and calcite precipitation. After 4 hours, Ca^+2^ concentrations approached 0.2 mM, suggesting calcite precipitation was near completion, and HCO_3_^−^ (63 mM) and CO_3_^−2^ (1.02 mM) concentrations approached pre-injection values. No significant changes in Ca^+2^, HCO_3_^−^, CO_3_^−2^, or Ω were observed between 4 and 22 hours. Small increases in ionic strength from near 500 mM to 700 mM occurred immediately after treatment following reductions in NH_4_^+^ and increases in Ca^+2^ (Fig. 5b). Solution pH also decreased from ≈8.6 to ≈7.5 and total alkalinity dropped from 150 to near 25 meq/L. The immediate drop in solution pH following cementation injections results from the generation of H^+^ during the consumption of CO_3_^−2^ from calcite precipitation. The reduction in total alkalinity reflects both the removal of residing NH_3_, HCO_3_^−^, and CO_3_^−2^ concentrations from solution replacement, the reduction of solution pH, and the consumption of CO_3_^−2^ during precipitation. After 4 hours, ionic strength values returned to pre-treatment values due to the production of NH_4_^+^ and consumption of Ca^+2^, solution pH approached ≈8.6, and total alkalinity returned to ≈150 meq/L. No significant differences were observed between 4 and 22 hours. After injection, urea concentrations increased from near 0 to 250 mM, total-NH_4_^+^ concentrations decreased from 425 to 100 mM, NH_4_^+^ concentrations decreased from 350 to 100 mM, and NH_3_ concentrations decreased from 75 to near 0 mM (Fig. 5c). Reductions in total-NH_4_^+^ and the presence of nearly all remaining total-NH_4_^+^ as NH_4_^+^ resulted from both solution replacement and the reduction in solution pH. After 2 hours, 150 mM urea was degraded and total-NH_4_^+^ increased by 175 mM. After 4 hours, urea concentrations were reduced to 50 mM and total ammonium further increased to 400 mM. Reductions in urea concentrations by ≈80% after 4 hours was comparable to urea degradation observed in stimulation columns on Day 9 (Fig. 4d). After 22 hours, urea concentrations approached 0 mM and total-NH_4_^+^ slightly decreased to ≈375 mM.

Aqueous chemical changes in the augmentation tank were similar to those in the stimulation tank, however, significantly faster ureolysis and calcite precipitation rates were observed. Immediately following the cementation injection, Ca^+2^ concentrations increased to near 200 mM and decreased to near 0 mM after only 2 hours (Fig. 5d). In addition, an initial concentration of 310 mM urea was degraded by ≈70% after 2 hours and ≈100% after 4 hours (Fig. 5f). This was ≈100 mM more urea degradation in the first 2 hours than in the stimulation tank. Higher ureolysis and calcite precipitation rates in the augmentation tank were consistent with observations from soil columns.

### Calcite precipitation kinetics

**Figure 6:**
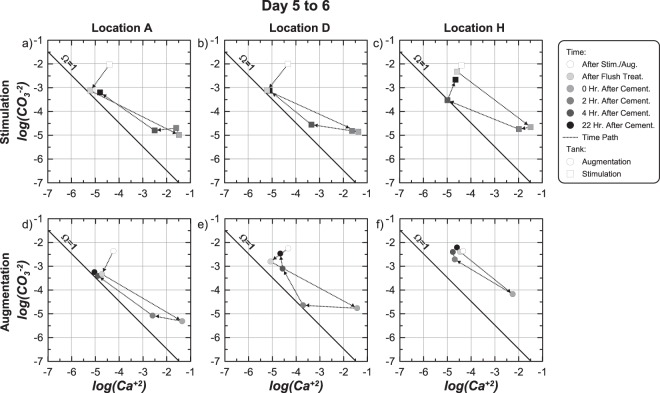
Activity ratio plots presenting log(CO_3_^−2^) versus log(Ca^+2^) values for sampling locations A, D, and H in the (**a**–**c**) stimulation and (**d**–**f**) augmentation tanks for 6 time points during the first cementation treatment on Day 5 to 6.

Figures 6a through 6f present log(CO_3_^−2^) versus log(Ca^+2^) values from solution measurements obtained at locations A, D, and H in stimulation and augmentation tanks at 6 time points during the flush and first cementation injection from Day 5 to 6. Measurements are presented on activity ratio diagrams where values plotting above, below, and on the Ω = 1 line are super-saturated, under-saturated, or at equilibrium with calcite, respectively. Locations A, D, and H were selected to highlight measurements at locations near injection W1 (Location A), production W2 (Location D), and at a location far from the injection alignment (Location H) (Fig. 1a). At locations A, D, and H in the stimulation tank (Fig. 6a–c), log(CO_3_^−2^) values were near −2.0 and log(Ca^+2^) values were near −4.5 following stimulation. Following the flush treatment, log(CO_3_^−2^) values reduced to near −3.1 at locations A and D, but remained higher near location H. At the same time, log(Ca^+2^) values decreased slightly at locations A and D, but remained near −4.5 at location H. Higher log(CO_3_^−2^) and log(Ca^+2^) values remaining near location H were attributed to the flush injection sequence used, which started with injections from W2 to W3.

In the augmentation tank immediately after augmentation (Fig. 6d–f), all locations had log(CO_3_^−2^) values near −2.25 and log(Ca^+2^) values near −4.5. Although these log(Ca^+2^) values were identical to those observed in the stimulation tank after stimulation, log(CO_3_^−2^) values in the stimulation tank were slightly higher due to four previous stimulation injections during which alkalinity was generated. Following the flush treatment in the augmentation tank, log(CO_3_^−2^) values were reduced to between −3.25 and −2.75 at locations A and D, but again remained higher at location H. Log(Ca^+2^) values also decreased slightly at locations A and D and remained constant at location H. In both tanks, large reductions in CO_3_^−2^ activities along the main well alignment of ≈1 order were obtained following the flush treatment. This resulted in significant reductions in solution super-saturation, with locations A and D achieving near equilibrium conditions prior to initiating cementation injections. This injection strategy was intended to reduce precipitation that would occur immediately at the solution mixing interface during the first cementation injection and potentially clog injection wells.

Immediately following the cementation injection, log(Ca^+2^) values increased and log(CO_3_^−2^) decreased at all locations in both tanks although at different magnitudes. Similar changes were observed at location A and D between tanks, however, lower log(Ca^+2^) and higher log(CO_3_^−2^) values observed at the more distant location H in the augmentation tank, suggested that ureolysis and precipitation reactions may have occurred more rapidly during solution transport than in the stimulation tank. 2 hours after injection, log(CO_3_^−2^) and log(Ca^+2^) values in the stimulation tank remained nearly unchanged from post-injection values, suggesting that neither significant urea degradation nor calcite precipitation had occurred. In the augmentation tank, however, log(Ca^+2^) values decreased dramatically in the first 2 hours at all locations. Faster precipitation observed in the augmentation tank is consistent with higher ureolysis rates observed from Day 5 to 6 (Fig. 4b). 4 hours after injection, reductions in log(Ca^+2^) values in the stimulation tank indicated that calcite precipitation was occurring, albeit at a much slower rate, with log(Ca^+2^) values between −2.5 and −3.5 at location A and D and larger reductions obtained at location H. During this same time, all locations in the augmentation tank approached pre-injection conditions, suggesting that calcite precipitation was largely complete. Between 4 and 22 hours, large decreases in log(Ca^+2^) values and increases in log(CO_3_^−2^) values occurred in the stimulation tank. In the augmentation tank, however, only small increases in log(CO_3_^−2^) were observed with nearly no changes in log(Ca^+2^) values. At all sampling locations in both tanks after 22 hours, log(Ca^+2^) values were between −4.5 and −5.1 and log(CO_3_^−2^) values were between −3.25 and −2.25. Although log(Ca^+2^) values were similar to those observed before injections, log(CO_3_^−2^) values were significantly lower than those remaining after biological treatments at location A and D due to the removal of CO_3_^−2^ during flush injections, large reductions in pH values from ≈9.5 to ≈8.6 following the start of calcite precipitation, and consumption of CO_3_^−2^ from calcite precipitation.

**Figure 7:**
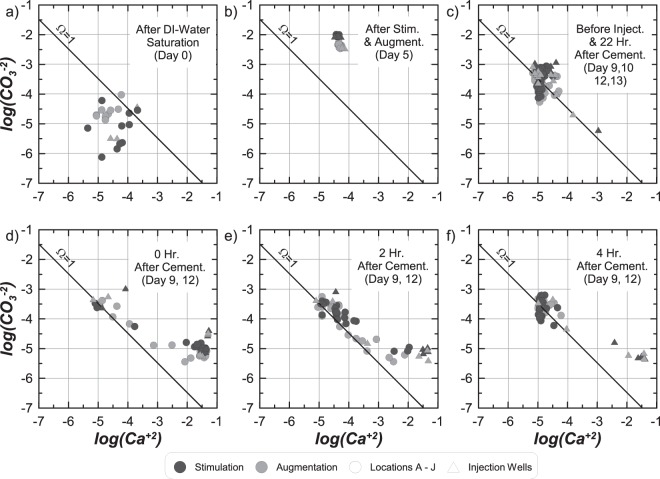
Activity ratio plots presenting log(CO_3_^−2^) versus log(Ca^+2^) values for all sampling locations in the stimulation and augmentation tanks: (**a**) after deionized water saturation on Day 0, (**b**) after stimulation and augmentation treatments, (**c**) before treatment injections and after 22 hr. treatment residence periods on Day 9, 10, 12, and 13, (**d**) immediately after cementation injections on Day 9 and 12, (**e**) 2 hours after cementation injections on Day 9 and 12, and (**f**) 4 hours after cementation injections on Day 9 and 12.

Figures 7a through 7f present log(CO_3_^−2^) versus log(Ca^+2^) values for all samples from both tanks obtained (a) after deionized water saturation on Day 0, (b) after all stimulation and augmentation treatments on Day 5, (c) immediately before cementation injections (after 22 hour residence periods) on Day 9, 10, 12, and 13, (d) immediately after cementation injections on Day 9 and 12, (e) 2 hours after cementation injections on Day 9 and 12, and (f) 4 hours after cementation injections on Day 9 and 12. Sampling intervals were selected to represent conditions during one cementation injection from W1 to W2 (Day 9 to 10) and W2 to W3 (Day 12 to 13). Following saturation with deionized water, almost all locations in both tank specimens were under-saturated with respect to calcite with log(CO_3_^−2^) values between −6.25 and −4.0 and log(Ca^+2^) values between −5.5 and −3.75 (Fig. 7a). After stimulation and augmentation, however, large increases in log(CO_3_^−2^) were observed to values near −2.0 and −2.5 in the stimulation and augmentation tanks, respectively, with log(Ca^+2^) remaining constant near −4.5 resulting in highly super-saturated conditions (Fig. 7b). During cementation, following a 22 hour residence period and immediately prior to a new cementation injection, most locations in both tanks were near equilibrium with log(CO_3_^−2^) values ranging from −4.0 to −3.0 and log(Ca^+2^) values ranging from −5.25 to −4.25 (Fig. 7c). For two well samples, however, log(Ca^+2^) values remained higher and log(CO_3_^−2^) values were lower suggesting that ureolysis and precipitation reactions were still likely ongoing. Immediately after cementation injections (Fig. 7d) most sampling locations obtained reductions in log(CO_3_^−2^) to values between −5.5 to −5.0 and increases in log(Ca^+2^) to values between −2.0 and −1.0 with no significant differences between tanks. Increases in log(Ca^+2^) values and reductions in log(CO_3_^−2^) values resulted from solution replacement, pH decreases, and CO_3_^−2^ consumption from precipitation. At locations further from the injection pathway, log(Ca^+2^) and log(CO_3_^−2^) values remained near pre-treatment conditions.

Two hours after cementation injections (Fig. 7e) reductions in log(Ca^+2^) and increases in log(CO_3_^−2^) values occurred at most locations. During precipitation, log(Ca^+2^) values first decreased at nearly constant log(CO_3_^−2^) values until the Ω = 1 condition was approached. Once near Ω = 1, further decreases in log(Ca^+2^) were not thermodynamically favorable without increases in log(CO_3_^−2^) from urea hydrolysis. Further reductions in log(Ca^+2^) values were therefore observed at conditions only slightly above Ω = 1. Four hours after injection (Fig. 7f) most locations were near equilibrium, however, log(Ca^+2^) and log(CO_3_^−2^) values remained near post-injection values at injection and production wells. Slower precipitation rates at well locations, which did not contain soil, suggested that the absence of soil surfaces may have significantly reduced ureolysis and precipitation rates. The close association of post-treatment conditions with Ω = 1 assuming a K_sp_ value of −8.48, suggests that solubility of bio-mediated calcite may not be significantly different than that for calcite minerals from the literature^29^. In addition, no distinguishable solubility differences between bio-cementation obtained with *S*. *pasteurii* and native ureolytic microorganisms could be discerned.

### Spatial distributions of calcite precipitation

**Figure 8:**
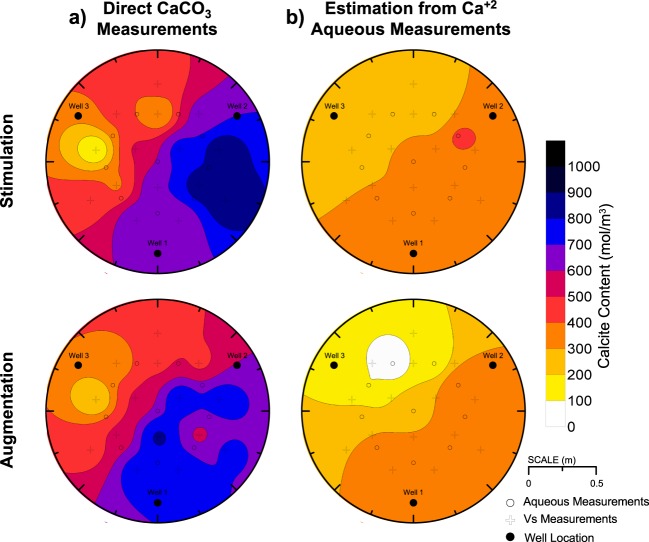
Inverse-distance interpolated spatial contours of soil calcite content from (**a**) direct chemical measurements and (**b**) aqueous Ca^+2^ estimations using post-injection measurements on Day 9 and 12.

Figure 8a presents inverse-distance interpolated spatial distributions of calcite content at mid-depth for both tanks determined from direct calcite measurements. Distributions of mid-depth calcite contents from direct measurements were similar between tanks with calcite contents ranging from 85 to 874 mol/m^3^ in the stimulation tank (average = 573 mol/m^3^) and 169 to 851 mol/m^3^ in the augmentation tanks (average = 573 mol/m^3^). Identical average calcite contents suggested that similar total masses of calcite had precipitated at mid-depth, albeit with small differences in distributions. In both tanks, the highest cementation occurred from W1 to W2 with the highest calcite contents measured closer to W1 in the augmentation tank and closer to W2 in the stimulation tank. In both tanks, the lowest calcite contents were measured near W3.

Figure 8b presents inverse-distance interpolated spatial distributions of mid-depth calcite contents for both tanks estimated from aqueous Ca^+2^ concentrations measured post-injection on Day 9 (W1 to W2) and Day 12 (W2 to W3). Calcite content distributions estimated from transported Ca^+2^ concentrations were calculated to quantify the amount of calcite that could be accounted for by assuming that all transported Ca^+2^ solution concentrations precipitated by the end of an injection period. Differences in calcite content spatial distributions between post-injection Ca^+2^ concentration based estimations (Fig. 8b) and direct measurements (Fig. 8a) were therefore used to infer the magnitudes of calcite that precipitated during solution injections. As shown, calcite content distributions estimated from post-injection Ca^+2^ measurements were similar between tanks, but were significantly lower than direct measurements suggesting that precipitation reactions occurring during solution transport were significant. Estimated calcite contents ranged between 72 and 442 mol/m^3^ in the stimulation tank (average = 314 mol/m^3^) and 44 to 415 mol/m^3^ (average = 265 mol/m^3^) in the augmentation tank. Although estimations and direct measurements were similar near W3, which never served as an injection well, post-injection Ca^+2^ based estimations were ≈500 mol/m^3^ lower than direct measurements from W1 to W2 and ≈200 to 300 mol/m^3^ lower than direct measurements from W2 to W3. Significant underestimation of calcite contents along both injection pathways using post-injection Ca^+2^ measurements suggested that significant precipitation occurred during transport along these alignments.

**Figure 9:**
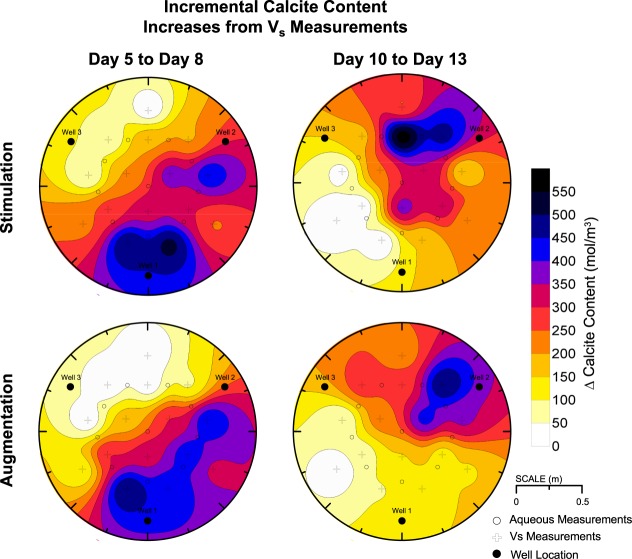
Inverse-distance interpolated spatial contours of incremental changes in soil calcite content estimated from V_s_ measurements obtained from Day 5 to 8 and Day 10 to 13.

Although temporal changes in calcite content distributions were not monitored directly, non-destructive shear wave velocity (V_s_) measurements were used to indirectly monitor changes in soil small-strain shear stiffness indicative of changes in soil calcite contents. Post-treatment V_s_ and direct calcite content measurements were used to develop relationships between V_s_ and soil calcite contents for respective tanks^40^. These relationships and V_s_ measurements at different times were then used to estimate calcite content distributions during treatments. Incremental changes in calcite contents were estimated for the first three days of cementation from Day 5 to 8 (W1 to W2 injections) and the last three days of cementation from Day 10 to 13 (W2 to W3 injections) (Fig. 9). Incremental calcite content distributions were similar between tank specimens for Day 5 to 8, however, more uniform calcite content increases were observed along the W1 to W2 alignment in the augmentation tank wherein all locations achieved calcite content increases greater than 400 mol/m^3^. In the stimulation tank, however, precipitation occurred at further locations near W3 with nearly all locations achieving increases greater than 50 mol/m^3^. More diffuse precipitation observed in the stimulation tank and greater localization of precipitation observed in the augmentation tank was attributed to differences in ureolysis (Fig. 4) and related precipitation rates (Fig. 5). Early during the cementation phase, faster ureolysis in the augmentation tank likely resulted in increased precipitation along the injection alignment, therefore limiting transport of Ca^+2^ to further regions near W3. In both tanks, estimated calcite content increases were greater than 450 mol/m^3^ near injection W1 and average estimated calcite content increases of 235 and 261 mol/m^3^ in the augmentation and stimulation tanks, respectively, were similar. Incremental calcite content distributions for Day 10 to 13 were more similar between tanks and reflected the change in injection direction. In both tanks, calcite content increases were greater than 450 mol/m^3^ near injection W2 and were ≈150 mol/m^3^ near W3. This calcite content increase near W3 was significantly lower than that observed near W2 from Day 5 to 8 and may have resulted from degradation of ureolytic activity in this region following limited solution flow from Day 5 to Day 10 and reductions in available nutrients and oxygen.

### Precipitate microstructure

**Figure 10:**
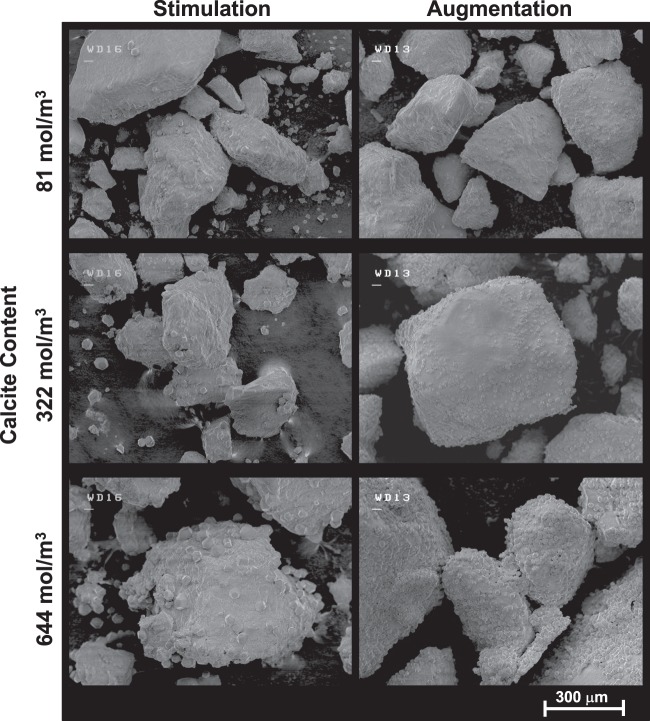
Scanning electron microscope (SEM) images at 100x of bio-cemented sand from augmentation and stimulation specimens at calcite contents of 81, 322, and 644 mol/m^3^.

Figure 10 presents SEM images of bio-cemented materials at similar calcite contents of ≈81, 322, and 644 mol/m^3^ from stimulation and augmentation columns. In general, fewer and larger calcite crystals were observed in stimulation specimens when compared to augmentation specimens at similar calcite contents. In addition, augmentation specimens appeared to be more uniformly coated with smaller crystals. At calcite contents near 644 mol/m^3^, crystals generally had diameters between 30 and 40 μm in the stimulation specimen and 5 to 10 μm in the augmentation specimen. The presence of larger crystals in the stimulation specimens is consistent with previous studies that have suggested that the formation of larger more well-structured crystals is promoted through slower precipitation rates^41^. Although ureolysis rates were comparable between stimulation and augmentation approaches after Day 9, initial nucleation of crystals occurring under slower precipitation rates may have influenced subsequent precipitation events and crystal growth. In both specimens, increases in crystal size and frequency were observed with increasing calcite content, suggesting that both continued growth of crystals and nucleation of new crystals occurred with subsequent treatments.

### Engineering improvement

While not discussed extensively in this manuscript, similar gradients of cementation and final engineering improvement were obtained in both tanks^22,40^. Cementation gradients were intentional and allowed for engineering improvements to be evaluated as a function of cementation level. Final calcite contents ranged from 0.5% to 5.3% by mass, shear wave velocities ranged between 131 and 967 m/s, and cone penetration resistances increased from between 3 and 5 MPa up to 32.1 MPa. At highly cemented locations near W1 in both tanks, soil shear wave velocities and cone penetration resistances increased by over 600% and 500%, respectively.

## Conclusions

The results of this study suggest that native ureolytic bacteria and augmented *S*. *pasteurii* can complete bio-cementation with similar changes in aqueous solution chemistry and post-treatment precipitate distributions at the meter-scale. However, it should be noted that recent findings^24^ strongly suggest that stimulated native strains may be dominating this process even in the augmented tank and columns by the end of the 8 day cementation phase. During stimulation, large increases in urea degradation from Day 3 to 4 indicated that native ureolytic microorganisms were active and completing urea hydrolysis in a spatially uniform manner prior to cementation. A complimentary soil column experiment was performed, which mimicked treatment conditions expected in the large-scale experiment, and ureolytic rates and cell density differences between approaches were examined. Results suggest that ureolysis rates within the stimulation tank were significantly lower than that in the augmentation tank from Day 5 to Day 9 despite having higher aqueous cell densities. Ureolysis rates in both specimens were similar and increased with additional cementation treatments at locations receiving concentrated cementation solutions. Slower precipitation rates observed early during the cementation phase in the stimulation tank resulted in calcite precipitation occurring at locations further from the injection well. In contrast, the augmentation tank had higher ureolysis and precipitation rates that resulted in greater localization of improvement along the main injection alignment. Monitoring of Ca^+2^ and CO_3_^−2^ changes indicated that precipitation reactions were largely complete in both tanks 4 hours after injections for all cementation treatments after Day 9. A comparison of measured and estimated calcite content distributions from direct measurements and transported Ca^+2^ concentrations suggested that precipitation reactions occurring during solution transport were significant in both tanks. Lastly, an examination of precipitation mediated by native ureolytic bacteria and augmented *S*. *pasteurii* showed that larger and fewer calcite crystals could be observed in stimulation specimens with a greater number of smaller crystals observed in augmentation specimens.

## Data Availability

The datasets generated during and/or analyzed during the current study are available from the corresponding author on reasonable request.
